# Longhorned beetle (Coleoptera: Cerambycidae) diversity in a fragmented temperate forest landscape

**DOI:** 10.12688/f1000research.1-25.v2

**Published:** 2013-03-14

**Authors:** Daniel M Pavuk, Andrea M Wadsworth

**Affiliations:** 1 Insect Ecology Laboratory, Department of Biological Sciences, Bowling Green State University, Bowling Green, OH, 43403-0208, USA

## Abstract

Longhorned beetles (Coleoptera: Cerambycidae) are an important component of temperate forest ecosystems.  We trapped longhorned beetles in forests in northwest Ohio during 2008 to test the hypothesis that larger forests have greater species diversity than smaller forests.  Large forests had a significantly greater cerambycid species richness than small forests (t = 3.16. P = 0.02), and there was a significant relationship between forest size and cerambycid species richness.

## Introduction

Longhorned beetles, or cerambycids, are important species in temperate forest ecosystems, due to their feeding impacts on trees. Many cerambycids feed on dead wood and therefore assist in the decomposition of dead trees in forest ecosystems. Saproxylic cerambycids (dead wood dependent) and other saproxylic beetles are thought to be useful indicators of forest biodiversity ^1^. We were interested in testing the hypothesis that larger forests have greater cerambycid species diversity than smaller forests in NW Ohio, a highly fragmented landscape in terms of forest ecosystems.

## Methodology

**Figure 1.**
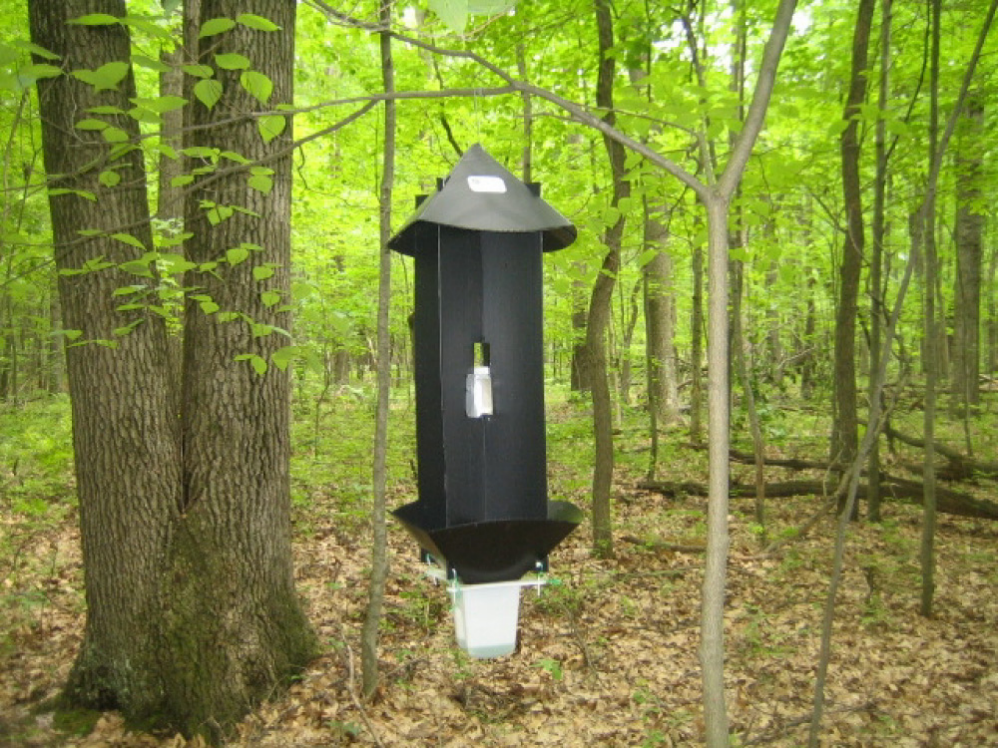
Intercept Panel trap used for capturing Cerambycid beetles.

- Three types of traps (Lindgren funnel trap, Intercept Panel trap, and Window trap) were set up in each of 8 forests in northwestern Ohio. 95% ethanol was used to attract beetles ( Figure 1– Figure 3).
- We started collecting beetles in early June, and we continued to collect them until early October ( Figure 4).
- We put the traps into 8 different forest areas. Four forests were large (>100 hectares) and four forests were classified as small (<20 hectares).

**Figure 2.**
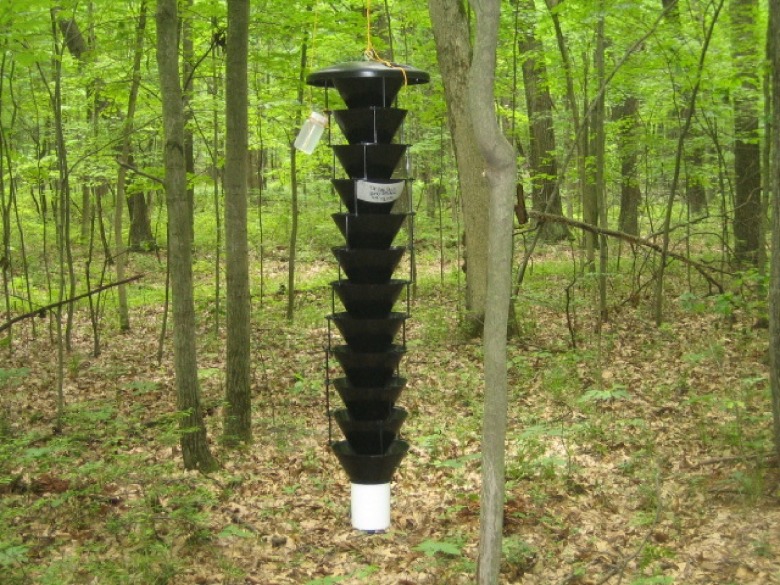
Lindgren Funnel trap used for capturing Cerambycid beetles.

**Figure 3.**
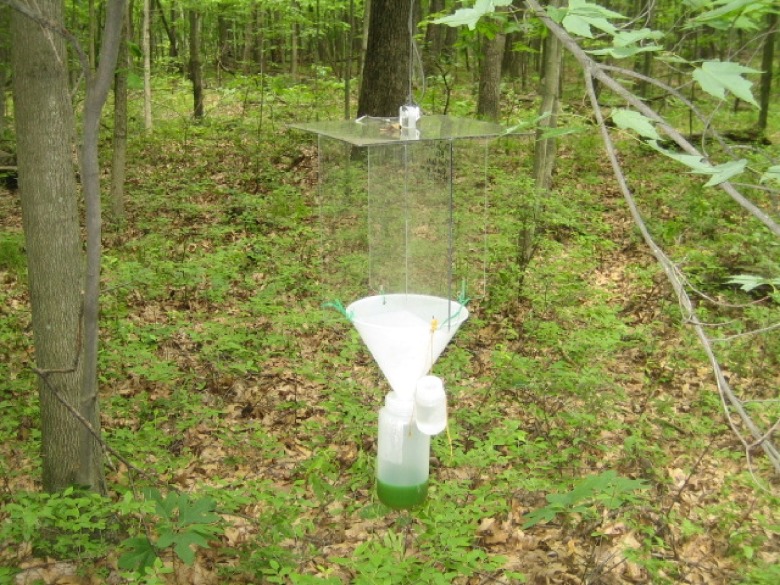
Window trap used for capturing Cerambycid beetles.

**Figure 4.**
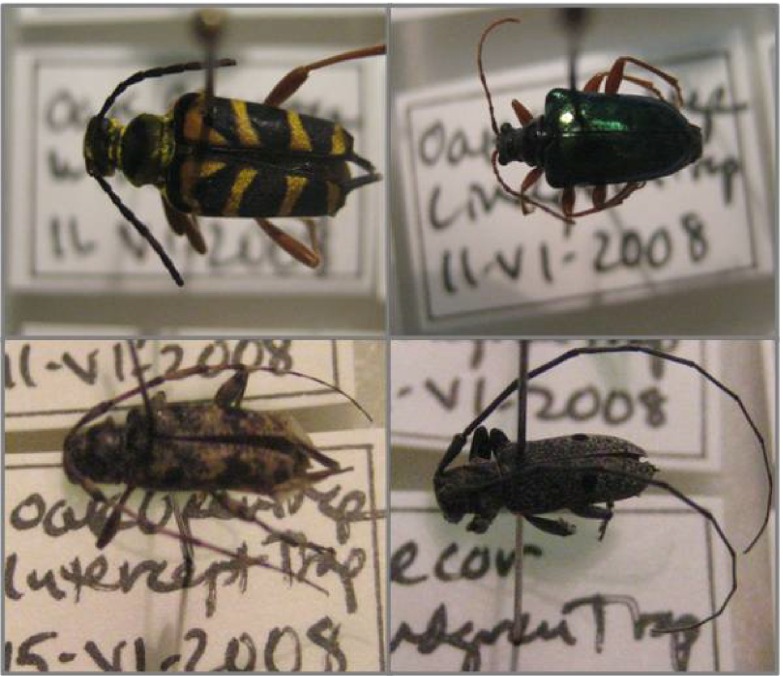
Examples of Cerambycids species that were caught in the 8 forests sampled. *Strophiona nitens* (top left), *Gaurotes cyanipennis* (top right) *Urographus fasciatus* (bottom left) and *Microgoes oculatus* (bottom right).

**Table 1.** Cerambycid (Coleoptera: Cerambycidae) Species Captured in Oak Openings Preserve, 2008 (Western Lucas County, OH). Total number of individuals of each species caught during the entire trapping period (early June to early October). Traps were positioned in the approximate center of each forest and all checked each week.

Cerambycid species	Number of individuals
*Strangalepta abbreviata* (Germar)	35
*Cyrtophilus verrucosus* (Olivier)	8
*Xylotechus colonus* (Fabricius)	8
*Clytus ruricolis* (Olivier)	5
*Gaurotes cyanipennis* (Fabricius)	3
*Neoclytus acuminatus* (Fabricius)	2
*Microgoes oculatus* (LeConte)	2
*Urographis fasciatus* (DeGeer)	2
*Pidonia ruficollis* (Say)	1
*Phymatodes testaceus* (Linnaeus)	1
*Sarosesthus fulminatus* (Fabricius)	1
*Strophiona nitens* (Forster)	1
*Urgleptes querci* (Fitch)	1
Total number of individuals (N)	70
Species richness (s)	13

**Table 2.** Cerambycid (Coleoptera: Cerambycidae) Species Captured in Secor Woods, 2008 (Northern Lucas County, OH). Total number of individuals of each species caught during the entire trapping period (early June to early October). Traps were positioned in the approximate center of each forest and all checked each week.

Cerambycid species	Number of individuals
*Clytus ruricola* (Olivier)	7
*Cyrtophilus verrucosus* (Olivier)	3
*Pidonia ruficollis* (Say)	3
*Gaurotes cyanipennis* (Fabricius)	2
*Microgoes oculatus* (LeConte)	1
*Psenocerus supernotatus* (Say)	1
Trachysida mutabilis	1
*Astylopsis collaris* (Haldeman)	1
Total number of individuals (N)	19
Species richness (s)	8

**Table 3.** Cerambycid (Coleoptera: Cerambycidae) Species Captured in Wildwood Preserve, 2008 (Northern Lucas County, OH). Total number of individuals of each species caught during the entire trapping period (early June to early October). Traps were positioned in the approximate center of each forest and all checked each week.

Cerambycid species	Number of individuals
*Strangalepta abbreviata* (Germar)	3
*Clytus ruricola* (Olivier)	2
*Gaurotes cyanipennis* (Fabricius)	2
*Microgoes oculatus* (LeConte)	2
*Cyrtophilus verrucosus* (Olivier)	1
*Analeptura lineola* (Say)	1
*Pidonia ruficollis* (Say)	1
Total number of individuals (N)	12
Species richness (s)	7

**Table 4.** Cerambycid (Coleoptera: Cerambycidae) Species Captured in Pearson Park, 2008 (Eastern Lucas County, OH). Total number of individuals of each species caught during the entire trapping period (early June to early October). Traps were positioned in the approximate center of each forest and all checked each week.

Cerambycid species	Number of individuals
*Clytus ruricola* (Olivier)	3
*Astylopsis macula* (Say)	2
*Urographis despectus* (LeConte)	1
*Cyrtophilus verrucosus* (Olivier)	1
*Psenocerus supernotatus* (Say)	1
Total number of individuals (N)	8
Species richness (s)	5

**Table 5.** Cerambycid (Coleoptera: Cerambycidae) Species Captured in Bradner Preserve, 2008 (Western Wood County, OH). Total number of individuals of each species caught during the entire trapping period (early June to early October). Traps were positioned in the approximate center of each forest and all checked each week.

Cerambycid species	Number of individuals
*Xylotrechus colonus* (Fabricius)	2
Total number of individuals (N)	2
Species richness (s)	1

**Table 6.** Cerambycid Cerambycid (Coleoptera: Cerambycidae) Species Captured in Carter Woods, 2008 (Central Wood County, OH). Total number of individuals of each species caught during the entire trapping period (early June to early October). Traps were positioned in the approximate center of each forest and all checked each week.

Cerambycid species	Number of individuals
*Heterachthes quadrimaculatus* Haldeman	4
*Xylotrechus convergens* LeConte	1
*Obrium maculatum* (Olivier)	1
Total number of individuals (N)	6
Species richness (s)	3

**Table 7.** Cerambycid (Coleoptera: Cerambycidae) Species Captured in Fuller Preserve, 2008 (Central Wood County, OH). Total number of individuals of each species caught during the entire trapping period (early June to early October). Traps were positioned in the approximate center of each forest and all checked each week.

Cerambycid species	Number of individuals
*Heterachthes quadrimaculatus* Haldeman	4
*Neoclytus acuminatus* (Fabricius)	3
Total number of individuals (N)	7
Species richness (s)	2

**Table 8.** Cerambycid (Coleoptera: Cerambycidae) Species Captured in BGSU-ENVS Woods, 2008 (Central Wood County, OH). Total number of individuals of each species caught during the entire trapping period (early June to early October). Traps were positioned in the approximate center of each forest and all checked each week.

Cerambycid species	Number of individuals
*Neoclytus acuminatus* (Fabricius)	2
*Sternidius variegatus* (Haldeman)	1
*Urgleptes signatus* (Fabricius)	1
*Anelaphus villosus* (Fabricius)	1
Total number of individuals (N)	5
Species richness (s)	4

**Table 9.** Shannon Diversity Index Values for Forest Study Sites.

Forest site	Shannon Index (H’ = -∑pilnpi)
Oak Openings	1.78
Secor Woods	1.81
Wildwood Preserve	1.86
Pearson Park	1.49
Bradner Preserve*	-
Carter Woods	0.87
Fuller Preserve	0.68
BGSU-ENVS Woods	1.33

*It is not possible to calculate a Shannon Diversity Index value for this site, because only one cerambycid species was caught.

## Results and discussion

**Figure 5.**
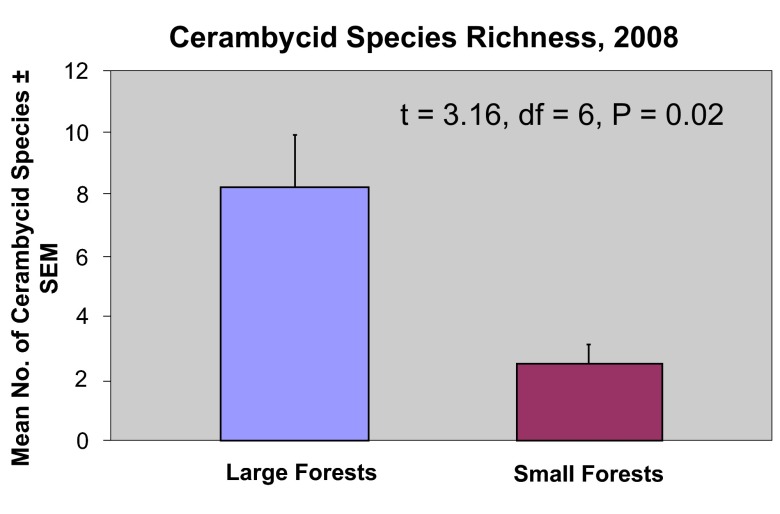
Comparison of Cerambycid species richness between large forests and small Forests. The four large forests were Oak Opening, Secor, Pearson, and Wildwood, and the four small forests were Bradner Preserve, Fuller Preserve, Carter Woods, and Environmental Studies Woods. The t-test was significant (t = 3.16, df = 6, P = 0.02).

**Figure 6.**
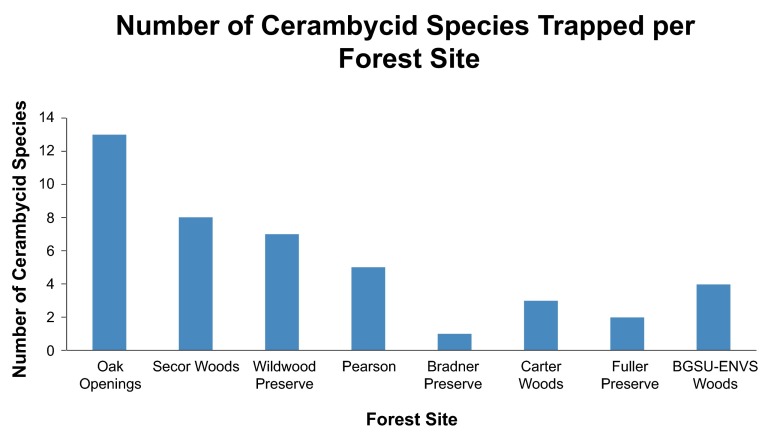
Total number of Cerambycid species caught in each forest during 2008. Oak Openings, Secor, Wildwood, and Pearson were the Large Forests, and Bradner Preserve, Carter Woods, Fuller Preserve, and ENVS Woods were the small forests.

**Figure 7.**
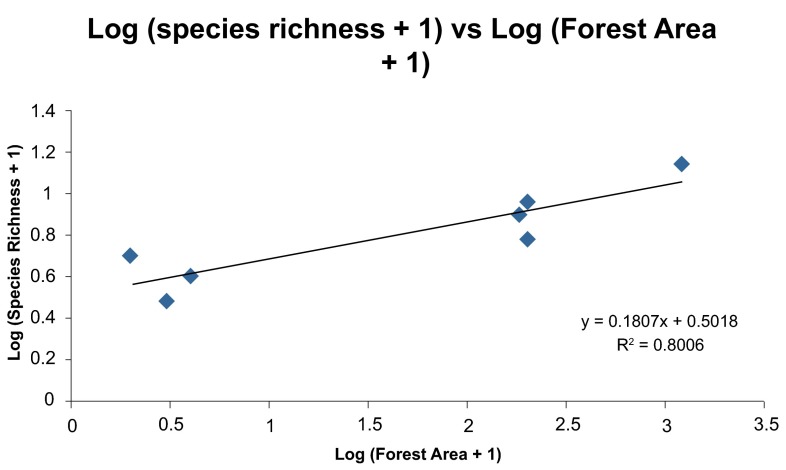
Regression of Log (Cerambycid Species Richness + 1) versus Log (Forest Area + 1).

- Large forests had greater cerambycid species richness than small forests. ( Figure 5, Figure 6).
  The larger forests had larger Shannon Diversity index values compared to the smaller forests. The calculated Shannon Diversity Index Values allows comparisons to other studies that have focused on cerambycid diversity in forest ecosystems ^1, 2^.
- Regression of log (species richness + 1) versus log (forest area + 1) indicated a strong linear relationship between cerambycid species richness and forest size (R ^2^ = 0.80). ( Figure 7). The large R2 value suggests that larger forests tend to have greater cerambycid species diversity than do smaller forests, perhaps due to greater amounts of resources for these beetles in the larger forests compared to the smaller ones.
- Future research should focus on the landscape matrix and degree of isolation of forests, especially isolation of smaller forests.
- Many other beetle species from other families were also captured (e.g., Elateridae, Curculionidae), so these data should also be examined.
