# Supplementary material for: Longhorned beetle (Coleoptera: Cerambycidae) diversity in a fragmented temperate forest landscape
Source: F1000Res. 2013 Mar 14;1:25. Originally published 2012 Oct 9. [Version 2] doi: 10.12688/f1000research.1-25.v2 (PMC3924947; doi:10.12688/f1000research.1-25.v2)
Supplement: Peer review history

# Peer review: Referee response for version 2

**Published:** 2014-01-20

**Authors:** Peter Silk

**Affiliations:** 1 Insect Chemical Ecology, Atlantic Forestry Centre, Fredericton, New Brunswick, Canada

I have read this submission. I believe that I have an appropriate level of expertise to confirm that it is of an acceptable scientific standard.

# Peer review: Referee response for version 2

**Published:** 2013-03-18

**Authors:** Patrick Tobin

**Affiliations:** 1 Ecology and Management of Invasive Species and Forest Ecosystems Unit,, USDA Forest Service, Morgantown, WV, USA

I have read this submission. I believe that I have an appropriate level of expertise to confirm that it is of an acceptable scientific standard.

# Peer review: Referee response for version 1

**Published:** 2012-10-17

**Authors:** Peter Silk

**Affiliations:** 1 Insect Chemical Ecology, Atlantic Forestry Centre, Fredericton, New Brunswick, Canada

I have read this submission. I believe that I have an appropriate level of expertise to confirm that it is of an acceptable scientific standard.

# Peer review: Referee response for version 1

**Published:** 2012-10-15

**Authors:** Patrick Tobin

**Affiliations:** 1 Ecology and Management of Invasive Species and Forest Ecosystems Unit,, USDA Forest Service, Morgantown, WV, USA

The overall merits of the study, general aspects of the experimental design, and hypothesis tested are interesting and conceptually sound.

The incredible amount of work involved in such a study is also recognized. A primary concern is that only one year of data was collected, which limits the interpretation of the findings. Measuring populations in the field over only one year, from which species richness was estimated, can be subject to annual variation that may lead to over- or underestimates of individuals in any given year. Moreover, some species of Cerambycidae may take more than one year to undergo a generation and thus these species could be undercounted. Regardless, the finding of increased richness with increased forest size confirms prior observations in related diversity studies. Also, the methods applied in this study are sound and thus provide a protocol in future endeavors.

One modification I would like to see in a revised manuscript is that instead of examining differences in richness between two categories (large versus small forests), I would prefer to see richness examined along a continuous measurement (i.e., the size of each forest), assuming that there is variation in sizes among the forests. In looking at Figure 6, I wonder if the respective area of each forest could explain the difference in species number. It also could be insightful to consider estimating diversity using established indices (e.g., Shannon, Simpson), which could help relate your work to other similar studies.

I have read this submission. I believe that I have an appropriate level of expertise to confirm that it is of an acceptable scientific standard, however I have significant reservations, as outlined above.
